# Objective validation of YouTube™ educational videos for the instruction of regional anesthesia nerve blocks: a novel approach

**DOI:** 10.1186/s12871-020-01084-w

**Published:** 2020-07-09

**Authors:** George L. Tewfik, Adam N. Work, Steven M. Shulman, Patrick Discepola

**Affiliations:** Department of Anesthesiology, Rutgers, New Jersey Medical School, 185 South Orange Ave., Newark, NJ 07103 USA

## Abstract

**Background:**

YouTube™ (“YouTube”) is often used as an educational tool to instruct anesthesia providers on regional anesthesia nerve blocks. However, there is no current objective standard to assess the educational quality of these user-uploaded videos. A new approach was used to objectively validate these videos by comparing them to high quality educational sources for the seven most commonly used nerve blocks.

**Objective:**

We sought to evaluate the educational quality of user-uploaded videos when compared to the highest quality anesthesia society websites (NYSORA, ACEP, USRA).

**Methods:**

After reviewing the instructional material available for the seven most frequently conducted nerve blocks on high-quality reference websites, we documented the educational characteristics present including such things as indications, volume, anatomy, etc. Next we reviewed the five most popular videos on YouTube for each block (by views) and documented the presence or absence of these educational characteristics.

**Results:**

Eighteen educational characteristics were documented in the “high-quality” anesthesia society reference material. Correlation was sought between this material and YouTube videos. Although there were varying degrees of correlation between the high quality sources and the videos, rarely did YouTube videos contain as high a percentage of these educational characteristics as the well-established sources. Some videos contained very few of these important educational characteristics.

**Conclusion:**

Although YouTube has been used an educational tool, we recommend that only high quality sources be used to teach or illustrate regional anesthesia nerve blocks.

## Background

As one of the most popular open source video content websites available today, YouTube has expanded from being a primarily entertainment website to an instrument for education and information sharing [1–4]. Numerous medical procedures and clinical skills instructional videos can be found quickly, changing the landscape of medical education [2, 4]. Students and providers alike are turning to social media platforms to augment classroom learning and refresh clinical skills [3, 4]. While access to these videos is nearly instantaneous, every piece of content must be used with the awareness that it is often neither peer reviewed nor certified for accuracy [2–4]. However, non-peer reviewed periodicals, websites, conferences and social media have been noted to be sources some healthcare professionals turn to prior to seeking out peer-reviewed material [2]. Nonetheless, many YouTube videos providing misinformation have been reported, leading to potential dangers to patients, and compromising providers’ credibility if used as the sole source of information [1, 5, 6].

Previous studies have analyzed instructional content for various specialties, the majority of which used subject matter experts. These are experts in “content analysis”, to grade the videos’ content based on predefined criteria [7–11]. Most concluded that, while convenient, YouTube content lacked the quality required to safely guide a provider through a specific procedure. Few studies have evaluated the value of regional anesthesia procedures posted to YouTube [9–11].

While others have evaluated large amounts of content posted to YouTube, we asserted that users are most apt to use the first few videos they come across, mostly corresponding to the “top hits,” or most viewed. Therefore, our aim was to design a system to objectively evaluate the 5 most viewed instructional videos for some of the most commonly performed regional anesthesia nerve block procedures.

## Methods

### Study design and setting

This observational retrospective study was conducted in the Department of Anesthesiology at Rutgers- New Jersey Medical School from July to August 2019. Our primary clinical site it University Hospital in Newark, NJ, and serves at New Jersey’s only fully public hospital, located in an urban center of nearly 300,000 residents.

### Study population and procedure

Four board certified anesthesiologists, each with at least 4 years of post-residency experience practicing regional anesthesia reviewed the content for ultrasound-guided nerve blocks of three well-vetted societies for regional anesthesia: The New York School of Regional Anesthesia (NYSORA )[12], American College of Emergency Physicians (ACEP )[13], and Ultrasound for Regional Anesthesia (USRA )[14] (Table 1). The following seven nerve blocks were reviewed: interscalene, supraclavicular, infraclavicular, axillary, femoral, popliteal and abdominus plane.

**Table 1:** Educational categories for instructional material of regional anesthesia nerve blocks. Column 1 displays the eighteen categories of educational characteristics, whose presence or absence was analyzed in NYSORA, ASRA, USRA and YouTube nerve block instruction. Column 2 is the percentage of instances in which the 35 analyzed YouTube videos (7 blocks with 5 videos each) met each educational characteristic

Indications	62.9%
Transducer position	94.3%
Goal	85.7%
Local anesthetic volume	71.4%
General considerations	94.3%
US anatomy	97.1%
Distribution of anesthesia	71.4%
Equipment	85.7%
Landmarks	97.1%
Patient positioning	91.4%
Technique	100.0%
Atlas picture of anatomy	48.6%
US picture of anatomy	97.1%
Picture of proper transducer placement	94.3%
Picture of proper needle placement	100.0%
Tips for preventing complications	88.6%
Guide for catheter placement	40.0%
References	17.1%

Eighteen key characteristics were determined to be present in nearly all of the educational material for these seven blocks. The decision was made to establish these eighteen characteristics as the control against which the Youtube videos would be evaluated (Table 1).

YouTube (www.youtube.com) searches were conducted for each nerve block (“interscalene nerve block”, “supraclavicular nerve block”, “infraclavicular nerve block”, “axillary nerve block”, “femoral nerve block” “popliteal nerve block” and “transversus abdominis plane block”). Each search returned between approximately 100 and 200 videos. The decision was made to limit evaluation to the top five videos by view count because after the 5th most popular video (by view count) the number of views often dropped off significantly below ~ 100 k views. In addition, it was believed by the researchers that the top five videos by view count would be the most utilized by potential learners, and that many users searching YouTube would not go past the second screen of search results to find an instructional video.

Next, search returns were organized using “filter”, then “sort by” and “view count.” The five videos for each block with the most views were evaluated. Inclusion criteria included content in the English language and ultrasound guided blocks (vs nerve stimulation only). Videos were excluded if they were not demonstrating an ultrasound guided block, were not in English, or were not relevant to the block/inappropriately titled or labelled. In each of the seven nerve block searches, the top video by view count had between 100,000 and 300,000 views, and the total of views for the top five videos was between 500,000 and 1,000,000 views total. With the significant drop-off for view count following the top five videos, this sample size was considered to be an appropriate representation of the quality of the most-reviewed reference videos.

Each YouTube video was analyzed for the presence or absence of each of these 18 characteristics, and this was recorded for further analysis. Characteristics were recorded as present only if the information contained was correct as determined by the attending anesthesiologists. However, after review, none of the information presented in these 18 categories, when found in the videos, was omitted due to inaccuracies.

## Results

The content of the three reference websites (NYSORA, ACEP and USRA) were analyzed for completeness using the categories outlined in Column 1 of Table 1. NYSORA had the most complete instructional content, recording 100% of the categories for each of the seven blocks. ACEP and USRA achieved means of 93.5 and 95.2%, respectively, of categories discussed. The TAP block was omitted for statistical calculations for ACEP, as they did not provide any instructional content for this particular block. “Indications” was the category missed most by USRA, with five out of seven blocks missing this content, while ACEP did not discuss “catheter placement” in any of its instructional videos.

Column 2 of Table 1 demonstrates the percentage of the analyzed YouTube videos (35 total videos from 7 blocks and 5 videos each) which contained each of the 18 educational characteristics, that were established as a control from NYSORA, USRA and ACEP. The least included category in the YouTube videos was “references”. Only 6 of the 35 (17.1%) videos evaluated by the research team referenced primary sources. “Guide for catheter placement” (40%) and “Atlas picture of anatomy” (48.6%) were the second and third least referenced categories included in all YouTube videos reviewed.

Table 2 compares and contrasts the differences in percentage of educational characteristics found in educational material between the 3 anesthesia societies (NYSORA, USRA and ACEP) compared to the YouTube videos. This is broken down by each of the individual 7 nerve blocks reviewed for this study. As is evident in the table, none of the reference material for the 7 blocks contains less than 94.4% of the educational categories in the anesthesia society reference material. Compare that to the YouTube videos, which range as low as 68.9% for the popliteal nerve block videos and no higher than 90.0% for the interscalene nerve block instructional videos. (Note: ACEP TAP block was omitted for statistical calculations, as they did not provide any instructional content for this particular block).

**Table 2:** Percent of educational categories referenced in educational material by nerve block in top 5 YouTube videos as determined by view count

	NYSORA, ACEP, USRA	YouTube
Interscalene	96.3%	90.0%
Supraclavicular	94.4%	82.2%
Infraclavicular	96.3%	86.7%
Axillary	96.3%	80.0%
Femoral	96.3%	73.3%
Popliteal	96.3%	68.9%
TAP	100.0%	77.8%

**Fig. 1:**
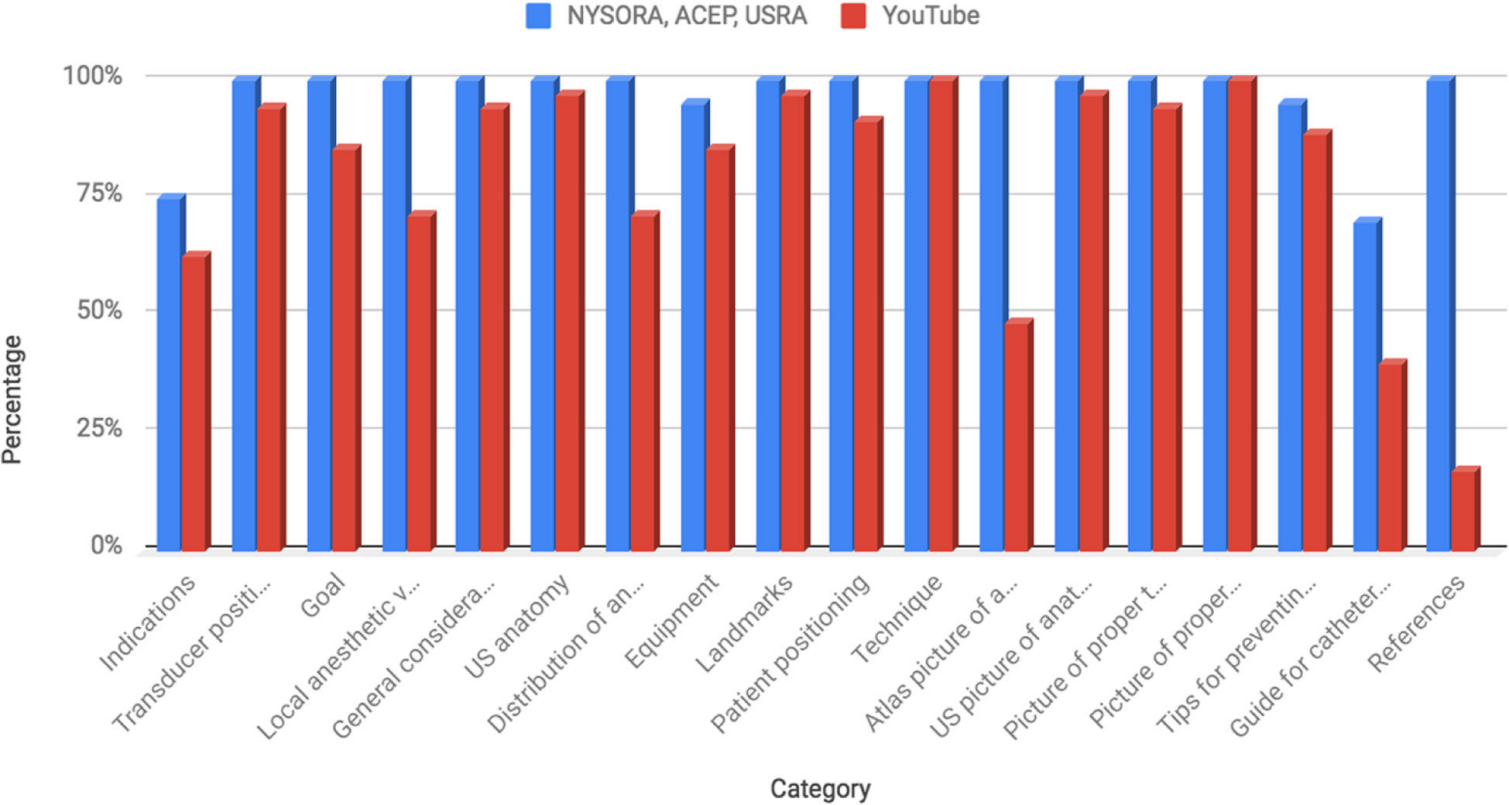
Comparison of percentage of educational characteristics present in the traditional online sources (NYSORA, ACEP and USRA) compared to the average of the YouTube videos

Overall, the regional anesthesia societies’ websites offered very thorough instruction for each nerve block. Figure 1 compares the percentage of educational characteristics present in the traditional online sources (NYSORA, ACEP and USRA) to the average seen in the YouTube videos. As demonstrated herein, in each of the 18 educational characteristics examined, there was a higher percentage of societal reference educational material that contained the material than was present in the YouTube videos analyzed.

**Fig. 2:**
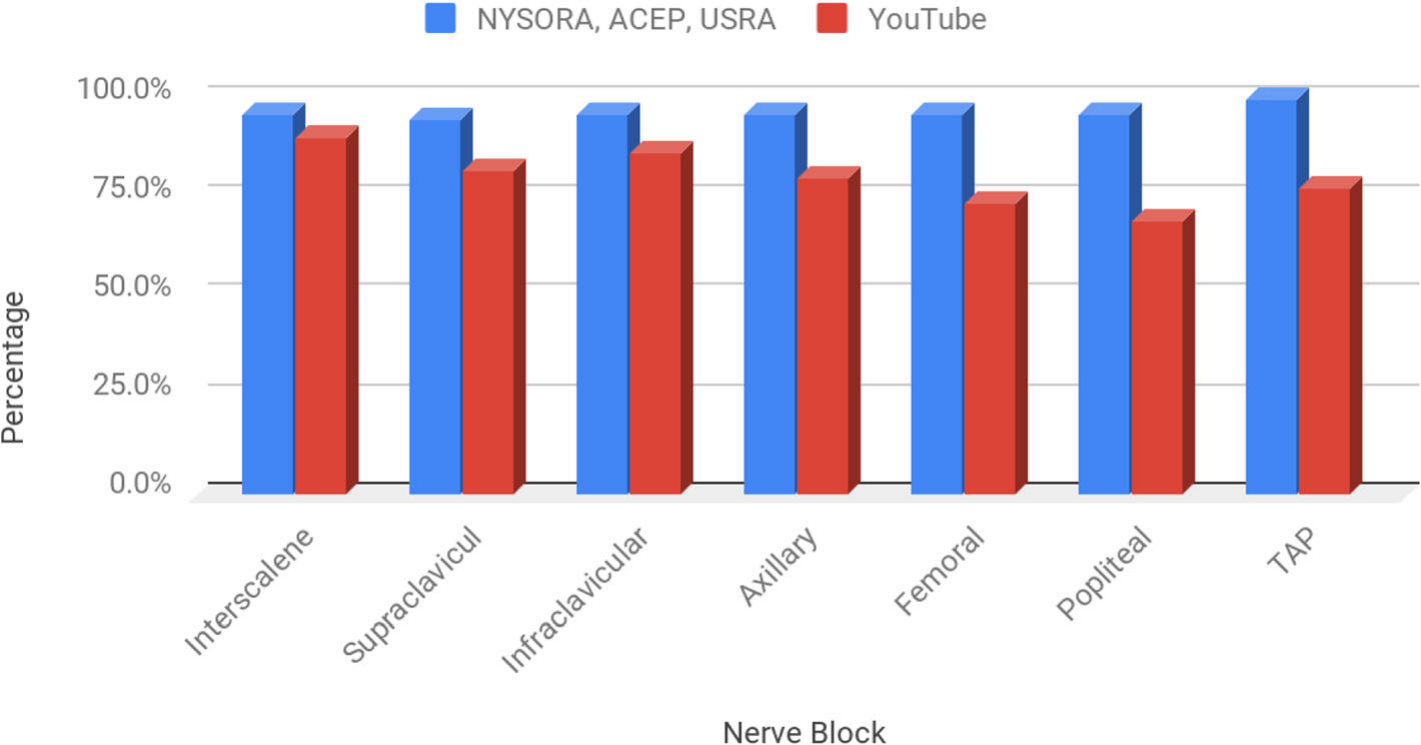
Percent of educational categories referenced in educational material by nerve block

Finally, Fig. 2 compares and contrasts the percentage of educational categories referenced in educational material by nerve block. For each of the seven nerve blocks, the educational material provided by NYSORA, ACEP and USRA contained higher percentages of the educational categories than the YouTube videos. Interestingly, the YouTube videos for the four brachial plexus blocks (interscalene, supraclavicular, infraclavicular and axillary) had a higher percentage of educational categories than other classes including lower extremity (femoral and popliteal) and TAP blocks.

## Discussion

Social media and internet sources for education are rising influences which have affected the study of medicine, especially in the last decade. These new tools may enhance professional networking and education, organizational promotion, patient care/education and public health programs [1, 5–8, 10, 11].

As the second most popular website in the world [15], with over one billion hours of video watched per day [16], YouTube is an extremely influential resource in entertainment and information gathering for millions of people. Not surprisingly, the site has become a frequently relied upon source of patient medical information gathering, especially by younger physicians, residents and students [17].

Given its popularity and global reach, YouTube has the potential to be an important educational tool for patients, conveying essential health information that could help decrease morbidity and economic burden of preventable diseases [18]. The challenge remains that, while abundant in quantity, the information available is often published by a non-vetted, non-peer reviewed source, and lacks the quality patients require.

There are numerous potential risks for patients and healthcare providers which can be caused by the dissemination of information of poor quality. These may cause damage to professional image, breaching patient privacy, violation of personal/professional boundaries and legal issues [1, 2, 6, 8, 9, 11–16]. Just as importantly, multiple studies evaluating the content of YouTube videos as a source of patient information across different specialties have found them to be of subpar quality and have questioned their usefulness in finding reliable and accurate information [19–22].

Just as YouTube has become a quickly turned to source of medical information for patients, medical students and providers are turning to its content more frequently [3, 4]. With younger generations of medical professionals having access to an exponentially increasing amount of content on YouTube and other social media platforms to augment their learning, caution must be taken prior to utilizing these sources. Urgent advocacy is needed for guidelines to prevent the risks presented by these new and alternate avenues of information dissemination and education [1, 7, 11, 16, 17].

Their content and publishers should be thoroughly vetted to ensure the information presented is complete and accurate. Other studies have evaluated YouTube content for physical exam skills, procedures, and surgeries and have found, similar to health informational videos accessed by patients, that their content is often questionable in quality and completeness [23–26].

Visual aids and hands on simulations can be invaluable tools in learning proper techniques in regional anesthesia. Recognizing the important role videos can play in augmenting regional anesthesia education, we set out to evaluate the content of the five most viewed videos for each of 7 ultrasound guided nerve blocks. While a few of the videos were very thorough, touching on nearly 90% of the 18 categories we designated as evaluation criteria, many were lacking a significant amount of information. The overall completeness of the YouTube videos was compared to the content published on the websites of three well respected authorities in regional anesthesia (NYSORA, ACEP and USRA) and noted to be significantly lacking. Figure 2 summarizes these findings, and raises troubling trends.

This study found that even among the five most viewed instructional videos per nerve block that there was a significant variation in completeness. Among all categories evaluated, “references” was missing from nearly 86% of YouTube videos. This is concerning given the fact that the site is an open source platform, allowing anyone to upload content without oversight. Without adequate sourcing of information, one cannot be certain of the accuracy of the information demonstrated to the learner. In contrast, the regional anesthesia society websites cited multiple references for each nerve block in question 100% of the time.

Whereas YouTube has the potential to be an invaluable tool for patients and providers, offering a plethora of extremely easily accessible content, it must be used in the proper manner in order to avoid receiving incomplete, and possibly inaccurate information. For students and young physicians, consulting the content published by the established authorities on regional anesthesia to learn foundational knowledge for each nerve block would be most advisable. YouTube likely cannot replace trusted sources for primary information, such as NYSORA, ACEP and USRA. However, there may be a role in consulting further video content after the provider has knowledge and experience with the procedure to evaluate different techniques or refresh their skills.

Anesthesiologists and physicians must remain resolute when evaluating educational sources for both their own education and disseminating information to patients or colleagues. Although more readily available than only a few years ago, clinical videos of high quality may be available only on sites that are behind a paywall. Whereas sites like those maintained by NYSORA, ACEP and USRA likely have editorial processes of high-quality, YouTube’s publishing process requires no editorial review except to ensure an absence of explicit, dangerous, violent, hateful, harassing, bullying, threatening or private material, or material that contains spam, scams or copyrighted material [27].

Past research has set forth potential guidelines for healthcare providers for the use of social media, which may be broadly applicable to the dissemination of online information [28]. Sample recommendations include ensuring content credibility by sharing only information from reputable sources and immediately refuting inaccurate information that a provider encounters [28].

Even more recent research has elucidated the possible effects of social media and internet sources of information as it related to the COVID-19 pandemic [29]. Fake news has been spread easily via social media as it relates to disease origins, manifestations, symptoms and treatments [29]. Social media has spread fake news leading to risky behaviors, such as taking unproven medications such as hydroxychloroquine [29].

When utilizing alternate education sources that are widely accessible, standards and guidelines may need to be established on both a local and national level. Further education may be incorporated for medical students, residents and even practicing anesthesiologists to be able to review, assess and differentiate the sources one uses on the Internet. Digital literacy should not be assumed, and may need to become a part of medical school and/or residency curricula.

Despite its limitations, however, YouTube will likely grow in influence in medical education. Advantages to publishing on YouTube include the speed of publishing new content and the widespread free access to videos and information. With growing Internet access throughout the world, especially on cell phones and mobile devices, practitioners who may have had limited access to information regarding new techniques and technologies, may be able to access an unending archive of instructional material on sites such as YouTube. This may lead to improved patient care and communication amongst medical professionals.

## Conclusion

As one of the most popular websites in the world, YouTube offers a wide range of video content, often including submissions for medical instruction. The proliferation of video content for medical education includes lessons on regional anesthesia nerve blocks. Although the potential for free, widespread and easily accessible educational material has a very high ceiling, care must be taken to ensure that this video content is of high caliber in order to ensure patient safety and the highest levels of professionalism.

This study sought to develop an objective system by which these free educational materials (in this case YouTube instructional videos for regional anesthesia nerve blocks) could be evaluated, and their quality validated prior to use by the public. Most of the educational videos reviewed on YouTube were of high quality, and contained many of the educational characteristics established from the reference materials. The purpose of this study was not only to compare the quality of videos on YouTube to high-quality reference material, but, more importantly, to attempt to develop an objective system for future evaluation and rating.

One potential solution to ensuring video content of the highest quality may be a rating system or a checklist to ensure certain educational content is present before uploading the material. Some of the videos uploaded to YouTube and evaluated in this study were from sources that produced content of higher quality including RAUKvideos, ThePainSource, Lecturio, Galusweegie, vidRASCI, BK Medical, NYSORA and ImedrxTV. However, without a rating system or system to evaluate the presence or absence of certain educational content, a simple search on YouTube does not elucidate the quality of the content, only the views and relevance based on titles and keywords. Herein we advocate for the implementation and use of an objective system to evaluate this widely-available educational content.

When compared to regional anesthesia societies’ website content, the content of the five most viewed instructional videos for each nerve block in question lacked important characteristics in multiple categories. Therefore, YouTube videos should likely not be used as primary sources when initially learning a nerve block; YouTube is not an effective substitute for textbook instruction, nor the high-quality educational material from anesthesia societies that specialize in regional anesthesia. However, these educational videos may be of value to augment already established knowledge for the experienced or learning provider wishing to refresh on skills or explore different techniques.

## Data Availability

Authors confirm that the data supporting the findings of this study are available within the manuscript and supplementary materials and all consent to publication.

## Abbreviations

NYSORA: The New York School of Regional Anesthesia
ACEP: American College of Emergency Physicians
USRA: Ultrasound for Regional Anesthesia
TAP: transversus abdominis plane
